# Comparative Efficacy of Photodynamic Therapy and Cold Knife Conization for Cervical High-Grade Squamous Intraepithelial Lesions

**DOI:** 10.3390/curroncol32110590

**Published:** 2025-10-22

**Authors:** Xiaoyun Wang, Yiquan Chen, Jianxia Huang, Qiong He, Jianwei Zhou

**Affiliations:** 1 Department of Gynecology, The Second Affiliated Hospital of Zhejiang University School of Medicine, No. 1511, Jianghong Road, Binjiang District, Hangzhou 310009, China; 2 Liangzhu Laboratory, The Second Affiliated Hospital of Zhejiang University, Zhejiang University, Hangzhou 310058, China

**Keywords:** photodynamic therapy, cervical conization, efficacy, HPV, cervical high-grade squamous intraepithelial lesions

## Abstract

(1) Background: Photodynamic therapy (PDT) using 5-aminolevulinic acid (5-ALA) offers a non-invasive alternative for managing cervical intraepithelial neoplasia. This comparative study assessed the efficacy and safety of 5-ALA PDT versus cervical conization in treating HSIL. (2) Methods: A total of 137 patients with HSIL and HPV infection, confirmed by pathological biopsy, were included. Based on patient preference and gynecological evaluation, participants were allocated into the PDT group and the CKC group. Follow-up assessments were conducted at 6 months post-treatment, involving HPV testing, cytology, and colposcopy. Additionally, any discomfort or side effects experienced during treatment were documented, along with a preliminary analysis of treatment costs and duration. (3) Results: The overall lesion remission rates were 88.0% for the PDT group and 89.6% for the CKC group, while HPV clearance rates were 80.0% and 82.7%, respectively. Although no statistically significant difference in efficacy was observed between the two groups, the PDT group exhibited a notably lower incidence of side effects. Nevertheless, the PDT group was associated with higher costs and longer treatment duration. (4) Conclusions: These findings indicate that 5-ALA PDT achieves comparable efficacy to CKC in the treatment of HSIL. Therefore, PDT may serve as a valuable therapeutic option for patients seeking fertility preservation or less invasive management strategies.

## Simple Summary

Cervical high-grade squamous intraepithelial lesions are primarily treated with surgical conization, which risks adverse events and impaired fertility. This study investigated a gentle, non-surgical alternative called photodynamic therapy, which uses a special light-activated gel to destroy abnormal cells without cutting. We compared photodynamic therapy directly against standard surgery in 137 patients. Our findings show that both treatments were equally effective at eliminating the lesions and the associated virus after six months. Crucially, photodynamic therapy demonstrated a significantly superior safety profile with fewer adverse events. Although associated with a longer treatment duration and higher cost, photodynamic therapy offers a safer option, especially for young women who wish to preserve their fertility. This study provides the necessary methodological details to facilitate future validation and clinical application, contributing to expanded and more personalized management strategies for cervical precancerous lesions.

## 1. Introduction

Approximately 20–30% of patients diagnosed with cervical HSIL may progress to cervical cancer if left untreated within 10 years [1]. Therefore, timely and effective treatment of HSIL is essential. Surgical excision, including CKC and the loop electrosurgical excision procedure (LEEP), is commonly employed to manage HSIL [2]. However, due to the high-temperature effect that can compromise surgical margins, LEEP often results in incomplete specimen integrity. By contrast, CKC is highly effective, allowing for the removal of clear margins and intact specimens, making it a widely preferred treatment option for HSIL [3].

Despite its effectiveness, surgical resection is associated with significant risks, such as major bleeding, infections, and obstetric complications, including cervical insufficiency, miscarriage, and preterm birth [4]. With the increasing emphasis on family planning and fertility preservation, there is a growing need for safer and more effective therapeutic options.

In recent years, PDT using the second-generation photosensitizer 5-aminolevulinic acid (5-ALA) has emerged as a promising non-invasive alternative for treating precancerous lesions of the female lower reproductive tract [5,6].

Early pioneering work, such as that by Bodner et al., suggested over a decade ago that PDT could achieve outcomes comparable to surgical interventions for cervical intraepithelial neoplasia (CIN) [7]. However, these earlier studies were often limited by smaller sample sizes and typically focused on CIN II. Consequently, high-quality evidence directly comparing PDT with the current gold standard, CKC, in a contemporary and larger cohort that includes high-grade lesions (HSIL/CIN3) remains valuable to solidify these findings. To address this question, the present retrospective study aimed to evaluate the efficacy of PDT in comparison with CKC for treating cervical HSIL.

## 2. Materials and Methods

### 2.1. Study Design and Ethical Considerations

Clinicopathological and follow-up data were collected for patients with cervical HSIL and persistent high-risk human papillomavirus (hr-HPV) infection who underwent PDT or CKC between January 2021 and December 2023. The study was approved by the institutional ethics committee before patient enrollment.

Patient allocation to the PDT or CKC group was based on patient preference following comprehensive, standardized counseling with a gynecologist, which detailed the procedures, benefits, and limitations of each treatment. To minimize selection bias, all patients had to meet the same strict eligibility criteria before being offered a choice. Furthermore, baseline characteristics were compared to ensure group comparability (Table 1), and statistical adjustments were employed in the analysis to control for potential confounders.

Exclusion criteria were: (1) cervical canal involvement; (2) inadequate colposcopy; (3) cervical surgery within the past year; (4) additional treatment after PDT or LEEP; (5) pregnancy or breastfeeding; (6) hypersensitivity to porphyrins or 5-aminolevulinic acid; (7) severe comorbidities requiring concurrent treatment, such as heart disease, arrhythmia, or active infection.

### 2.2. Treatment Procedure

#### 2.2.1. 5-ALA PDT

A 20% solution of 5-ALA was prepared by mixing 354 mg of ALA powder (Shanghai Fudan–Zhangjiang BioPharmaceutical Co., Ltd., Shanghai, China) with 1.5 mL of thermogel. The cervix and vagina were cleaned with sterile 5% sodium chloride before applying sterile cotton sheets soaked with the ALA solution to the cervical surface and canal. A condom filled with medical gauze was inserted to ensure optimal contact between the cotton sheets and the cervical tissue.

After a 4 h incubation period, light irradiation of the cervical surface was performed using a 635 nm LED light source with a dose of 100 J/cm^2^. Cervical canal illumination was performed using a 635 nm diode laser (LD600-C; Wuhan Yage Optoelectronics Co., Ltd., Wuhan, China) with a cylindrical diffuser and the same light dose. Six treatment sessions were given every 1–2 weeks. Discomfort and adverse effects were recorded during treatment.

#### 2.2.2. CKC

All CKC procedures were conducted in an operating room under intravenous anesthesia by experienced gynecologists. Iodine staining was used to delineate the extent of the lesion, and conization involved removing the complete transformation zone and part of the cervical canal above the squamocolumnar junction (SCJ). Depth of resection varied by transformation zone type: 7–10 mm for type 1, 10–15 mm for type 2, and 15–25 mm for type 3. Patients with type 3 zones were excluded for consistency with the PDT group. Hemostasis was achieved using modified Sturmdorf sutures, and iodophor-soaked strips were applied for 24 h. All specimens were reviewed by experienced gynecological pathologists. Discomfort and side effects were monitored throughout the treatment.

### 2.3. Follow-Up and Outcome Assessment

All patients underwent follow-up evaluations at 6 months and 12 months post-treatment, including HPV-DNA testing, cervical cytology, and colposcopy-directed biopsy. HPV-DNA was detected using probes (Jiangsu Jianyou Medical Technology Co., Ltd., Danyang, China) targeting 24 HPV subtypes (low-risk: 6, 11, 42, 43, 44, 81, 83; high-risk: 16, 18, 31, 33, 35, 39, 45, 51, 52, 53, 56, 58, 59, 66, 68, 73, 82). Cytology was assessed via ThinPrep cytology tests (TCT) with standard terminology: negative for intraepithelial lesion or malignancy (NILM), atypical squamous cells of undetermined significance (ASCUS), low-grade squamous intraepithelial lesion (LSIL), and HSIL. Histological evaluations adhered to the 2014 World Health Organization Classification.

Cure was defined as the absence of detectable HPV-DNA, negative TCT results, and no HSIL on colposcopy-directed biopsy. Remission referred to the downgrade of HSIL to LSIL, irrespective of HPV and TCT results. The pathological efficiency rate included cases of cure and remission, while the HPV-negative rate accounted for complete clearance of all HPV subtypes. Persistent or partially negative HPV results were categorized as HPV positive.

### 2.4. Statistical Analysis

All data were analyzed using IBM SPSS Statistics Version 24.0. Results were expressed as counts and percentages (N [%]), and chi-square tests were employed for group comparisons. Differences were considered statistically significant at *p* < 0.05.

The odds ratio (OR) and 95% confidence interval (CI) were used to evaluate the relationship between related factors and outcome.

## 3. Results

### 3.1. Baseline Characteristics

**Figure 1:**
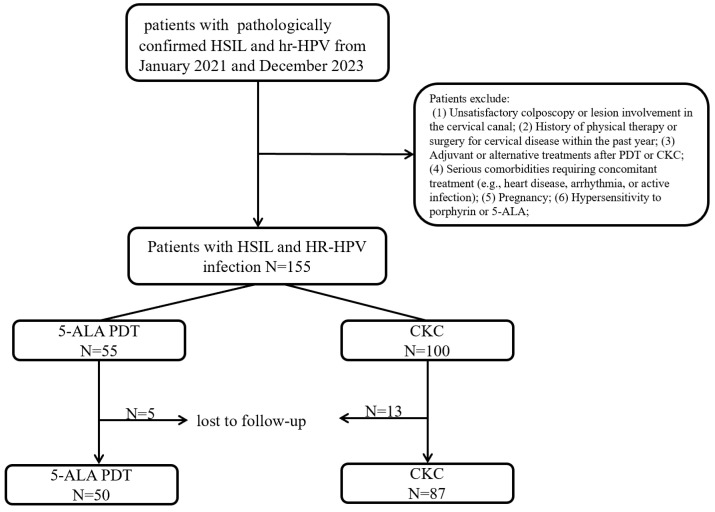
The follow-up flowchart of the study subjects.

The 155 patients were divided into the PDT group (55 patients) and the CKC group (100 patients) due to their willingness and the advice of gynecologists. Due to loss to follow-up (5 patients in the PDT group and 13 patients in the CKC group), 137 patients were ultimately included in the analysis (PDT group: N = 50; CKC group: N = 87). The trial flowchart is presented in Figure 1. The mean age of patients in the PDT group was 30.9 years (range: 25–48 years), compared to 36.0 years (range: 24–65 years) in the CKC group. No statistically significant differences were observed between the two groups in terms of age, HPV infection status, TCT results, or lesion characteristics (Table 1).

### 3.2. Comparison of HPV-Negative Rates

At the 6-month follow-up after treatment, 40 of 50 patients (80.0%) in the PDT group achieved HPV negativity. Among the remaining 10 patients, 6 exhibited partial clearance of HPV infection. Subgroup analysis based on HPV typing revealed an HPV16/18 negative rate of 80.0% (24/30) and a non-16/18 HPV negative rate of 80% (16/20). Analysis by lesion severity showed that the HPV-negative rate was 84.1% (37/44) for CINII lesions and 50% (3/6) for CINIII lesions. In the CKC group, 72 of 87 patients (82.7%) achieved HPV negativity, while 15 patients remained HPV-positive, among whom 8 exhibited partial clearance of HPV infection. Subgroup analysis based on HPV typing revealed an HPV16/18 negative rate of 82.2% (37/45) and a non-16/18 HPV negative rate of 83.3% (35/42). Analysis by lesion severity showed that the HPV-negative rate was 84.6% (55/65) for CINII lesions and 77.3% (17/22) for CINIII lesions. No significant differences were observed in HPV-negative rates between the two groups, either in terms of HPV typing or lesion severity (80.0% vs. 82.7%, *p* > 0.05; Table 2).

### 3.3. Comparison of the Lesion Effective Rates

Cure was defined as the simultaneous clearance of HPV and lesion regression. At 6 months, 84.0% of patients in the PDT group and 85.0% in the CKC group were considered cured. The cure rates between the two groups did not differ significantly (*p* > 0.05).

Assessment also revealed that 6.0% of patients in the PDT group and 4.6% in the CKC group experienced lesion downgrading. The overall lesion remission rate was slightly higher in the CKC group compared to the PDT group (89.6% vs. 88.0%), but this difference was not statistically significant (*p* > 0.05). Importantly, no cases of lesion progression were observed in either group during the follow-up period (Table 3). Notably, during the follow-up period, no patients in either group experienced reinfection with the same HPV type or lesion recurrence, and no lesion progression was observed.

Although lesion progression was absent, lesion persistence was identified in 6 patients in the PDT group and 9 in the CKC group. Among the PDT group, two patients (aged 30) with pre-treatment multiple high-risk HPV infections (e.g., HPV 18/31/39/51/58/82 and HPV-58) underwent laser therapy. Two others (aged 31 and 34) with pre-treatment HPV 6/33/18 and HPV-16/59 infections underwent LEEP. Another two patients (aged 24 and 30) with pre-treatment multiple infections opted for repeated PDT combined with laser ablation due to thicker lesions. In the CKC group, 6 patients exhibited multiple HPV infections, while 3 had single infections with HPV-16 or HPV-18. Of these, 4 patients chose PDT, 2 underwent laser therapy, 1 underwent repeated CKC, and 2 patients over 50 opted for total hysterectomy.

**Figure 2:**
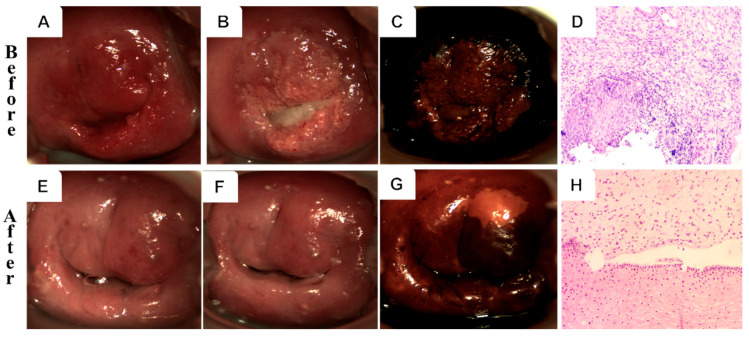
Colposcopy and histology of a 32-year-old HPV-16/58-positive patient before (upper row) and after (lower row) PDT. (**A**,**E**) Gross view; (**B**,**F**) acetic acid test; (**C**,**G**) iodine staining; (**D**,**H**) H&E (10×).

**Figure 3:**
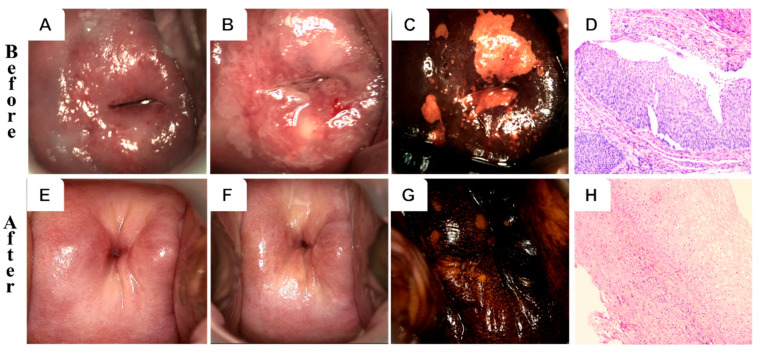
Colposcopy and histology of a 55-year-old HPV-58 positive patient before (upper row) and after (lower row) CKC. (**A**,**E**) Gross view; (**B**,**F**) acetic acid test; (**C**,**G**) iodine staining; (**D**,**H**) H&E (10×).

Figure 2 and Figure 3 illustrate representative colposcopic and histopathological findings before and after treatment in both groups. A 32-year-old patient in the PDT group with HPV 16/58 infection exhibited thick acetic acid staining and positive iodine staining, consistent with HSIL/CIN II. Histological examination (H&E staining) revealed squamous epithelial hyperplasia with lymphocyte infiltration. Post-treatment evaluation showed a normal cervix and re-epithelialization, achieving complete cure. In contrast, a 55-year-old patient in the CKC group with HPV 58 infection displayed HSIL/CIN III, including thick acetic acid staining and positive iodine staining. Histology revealed a proliferating cell compartment and koilocytosis. After treatment, the cervix appeared normal with chronic cervicitis detected histologically. However, structural changes and scar formation were noted despite achieving complete cure.

### 3.4. Logistic Regression Analysis of Factors for Lesion Remission and HPV Clearance

The association between baseline clinical characteristics and therapeutic efficacy was analyzed between two groups. Multiple HPV infections were found to be significantly associated with both the effectiveness of PDT and HPV clearance rates, while no significant associations were observed with other variables. These findings indicate that multiple HPV infections are a significant predictor of suboptimal PDT treatment outcomes at the 6-month follow-up (*p* < 0.05, OR < 0.1; Table 4).

### 3.5. Comparison of Side Effects

**Figure 4:**
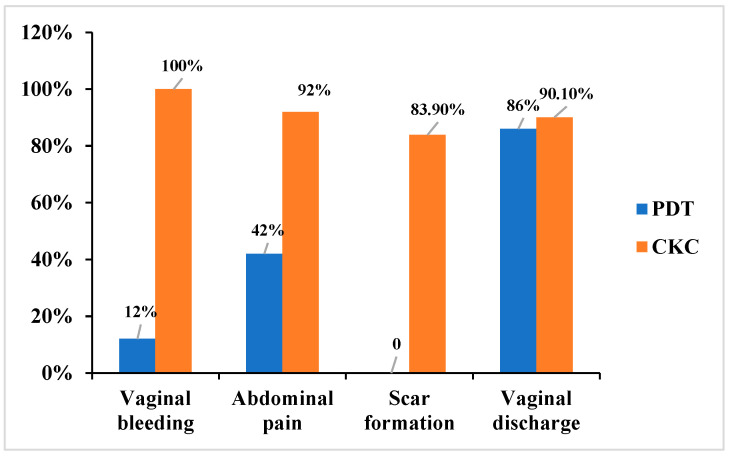
Adverse reactions of ALA-PDT and CKC groups.

The incidence rates of vaginal bleeding (12% vs. 100%), abdominal pain (42% vs. 92%), and scar formation (0 vs. 83.9%) were significantly lower in the PDT group compared to the CKC group (*p* < 0.05). In contrast, the frequency of increased vaginal discharge during the short-term follow-up period was similar between the two groups (Figure 4).

In the CKC group, 100% of patients experienced vaginal bleeding, 92.0% reported abdominal pain, and 83.9% exhibited scar formation. These rates were significantly higher than those observed in the PDT group (*p* < 0.01). Among CKC patients, 4 presented to the emergency department 10–14 days post-surgery due to secondary hemorrhage, which required vaginal gauze compression for 24 h. Additionally, 2 patients developed cervical adhesion stenosis requiring reoperation, and 1 non-nulliparous patient experienced cervical canal shortening and cervical insufficiency following surgery (Figure 3).

In contrast, side effects in the PDT group were notably milder. Only 12.0% of patients experienced minimal vaginal bleeding, and 42.0% reported abdominal pain. These side effects were generally tolerable and resolved within 3–7 days post-treatment. Importantly, for patients with cervical HSIL, PDT did not adversely affect cervical structure or function, as demonstrated in Figure 2.

In addition, 3 patients successfully delivered vaginally following PDT treatment. To date, no cases of adverse effects on pregnancy or delivery attributable to cervical lesions have been identified.

## 4. Discussion

HSIL are premalignant conditions, histologically classified as CIN2 and CIN3. Without timely intervention, HSIL may progress to cervical cancer [8]. Historically, surgical excision techniques, including CKC, laser conization/ablation, and LEEP, have been considered standard treatment options for HSIL. Among these, CKC remains the preferred method due to its ability to ensure clear resection margins and its high cure rate [9]. However, CKC is associated with significant complications, including hemorrhage, infection, cervical deformation, and cervical canal adhesion. These adverse effects can increase the risk of obstetric complications, such as miscarriage and preterm birth [10]. With an increasing emphasis on fertility preservation, particularly among younger women, there is a pressing need for non-invasive treatment options that minimize these risks while maintaining efficacy.

5-ALA is a precursor to the photosensitizer protoporphyrin IX (PpIX) and has been successfully utilized in the PDT procedures for conditions such as cervical condyloma acuminatum, CIN, and other gynecological disorders [11,12,13]. Our previous research demonstrated that PDT achieved a pathological regression rate of 90.1% for LSIL and an HPV clearance rate of 81.8% [14]. Currently, the expert consensus in Chinese obstetrics and gynecology identifies PDT as a viable treatment option for cervical HSIL [15]. In prior studies comparing PDT with LEEP, PDT demonstrated comparable or superior efficacy in treating HSIL and clearing HPV infections. For instance, a study reported cure rates of 88.1% for PDT and 70.0% for LEEP at 6 months post-treatment (*p* < 0.05) [16]. These findings underscore the efficiency and non-invasiveness of PDT. Although early studies have demonstrated the potential of PDT [7], high-quality direct comparisons with CKC, the established gold standard, particularly within larger and more representative cohorts that include a substantial number of CIN3 patients, remain limited. This study was specifically designed to address this critical gap in the current evidence base.

Our findings align with those of Tang et al., who reported a lesion regression rate of 77.78% among 99 HSIL patients treated with PDT [17]. Similarly, a single-center cohort study observed a 92.0% lesion regression rate for HSIL patients receiving PDT [18]. In our study, the overall efficiency rates for the PDT and CKC groups were 88.0% and 89.6%, respectively. Furthermore, the HPV clearance rates were 80.0% for the PDT group and 82.7% for the CKC group, while the complete cure rates were 84.0% and 85.0%, respectively. These results suggest that the efficacy of PDT is comparable to CKC in treating cervical HSIL, corroborating previous reports [19,20].

Further stratified analyses based on HPV types and lesion severity revealed no statistically significant differences in HPV-negative rates or lesion effective rates between the two groups. Multivariate analysis failed to identify any significant correlations between age, transformation zone type, lesion severity, HPV type, and other related factors with either HPV-negative rates or lesion effective rates. This lack of correlation may be attributable to the limited sample size and single-center nature of the study. The progression of HSIL and HPV clearance may be influenced by multiple factors, including host immune response, and patient-specific characteristics. Furthermore, emerging evidence indicates that various trace elements and micronutrients may exert a protective effect against cervical cancer by modulating key stages in the natural history of HPV infection, cervical dysplasia development, and progression to invasive disease [21].

Importantly, 5-ALA PDT demonstrated a favorable safety profile. Cervical structure and function remained intact following treatment, with no cases of adverse tissue reactions or photosensitization reported. Furthermore, 3 women successfully achieved vaginal delivery following PDT treatment. By contrast, CKC was associated with significant side effects, including heavy bleeding, scarring, cervical adhesions, and cervical canal shortening. These complications not only necessitate additional interventions but also pose challenges to subsequent pregnancy and complicate postoperative follow-up and retreatment for residual or recurrent lesions [22,23]. Notably, PDT offers the advantage of repeatability in cases of HPV reinfection or lesion recurrence [24].

Although this study has several strengths, it is subject to certain limitations. It is a retrospective analysis with a non-randomized design, limited follow-up duration, and a small sample size in the CIN3 subgroup. Future multi-center, large-sample prospective randomized controlled trials with extended follow-up periods are warranted to further validate the clinical efficacy of photodynamic therapy.

## 5. Conclusions

In conclusion, the findings of this study confirm and strengthen the existing evidence that 5-ALA PDT achieves similar short-term efficacy to CKC in treating cervical HSIL, with the added benefit of fewer adverse effects. While this core concept was previously suggested, our work substantiates it with a larger cohort, the inclusion of CIN3 patients, and a detailed side-effect profile. These results support 5-ALA PDT as a valuable non-invasive therapeutic alternative, particularly for young women or those seeking to preserve fertility.

## Figures and Tables

**Table 1:** Clinical characteristics of the study population.

Clinical Characteristics	PDT (N = 50)	CKC (N = 87)	*p* Value
Age			0.08
<40	41	58	
20–29	24	20	
30–39	17	38	
≥40	9	29	
Median (range)	30.9 (25–48)	36.0 (24–65)	
HPV genotype			0.45
16/18	30	45	
Non-16/18	20	42	
Single	25	43	1
Multiple	25	44	
TCT			0.34
NILM	16	20	
ASC-US	15	28	
LSIL	15	20	
HSIL	4	19	
Transformation Zone			0.81
TZ I	30	49	
TZ II	18	30	
TZ III	2	8	
Lesion characteristics			0.10
CIN II	44	65	
CIN III	6	22	

**Table 2:** Comparison of HPV-negative rates between two groups at 6 months follow-up.

HPV		The 6th Month (95%CI)	
	PDT	CKC	*p* Value
Negative rates	80.0% (66.3–90.0%)	82.7% (73.2–90.0%)	0.86
HPV genotype			
HPV16/18	80.0% (61.4–92.3%)	82.2% (67.9–92.0%)	1
non-HPV16/18	80.0% (56.3–94.3%)	83.3% (68.6–93.0%)	1
Lesion severity			
CIN II	84.1% (69.9–93.4%)	84.6% (73.5–92.3%)	1
CIN III	50.0% (11.8–88.2%)	77.3% (54.6–92.2%)	0.42
Positive			
Partial-negative	12.0% (4.5–24.3%)	9.2% (4.1–17.3%)	0.82
Persistence	8.0% (2.2–19.2%)	8.1% (3.2–15.9%)	1

**Table 3:** Comparison of the lesion remission between two groups at 6 months follow-up.

Lesion		The 6th Month	(95%CI)
	PDT	CKC	*p* Value
Effective rates	88.0% (75.7–95.5%)	89.6% (81.3–95.2%)	0.99
Cure	84.0% (70.9–92.8%)	85.1% (75.8–91.8%)	1
Remission	6.0% (1.3–16.5%)	4.6% (1.3–11.4%)	1
HPV genotype			
HPV16/18	90.0% (73.5–97.9%)	91.1% (78.8–97.5%)	1
non-HPV16/18	85.0% (62.1–96.8%)	88.1% (74.4–96.0%)	1
Lesion severity			
CIN II	86.4% (72.6–94.8%)	89.2% (79.1–95.6%)	0.88
CINIII	100.0% (54.1–100%)	90.9% (70.8–98.9%)	1
Ineffective			
Persistence	12.0% (4.5–24.3%)	10.3% (4.8–18.7%)	0.99
Progression	0 (0.0%)	0 (0.0%)	

**Table 4:** Logistic regression analysis of factors for lesion remission and HPV clearance.

	The 6th Month							
Clinical Characteristics	PDT				CKC			
	Lesion Remission		HPV Clearance		Lesion Remission		HPV Clearance	
	OR (95%CI)	*p*	OR (95%CI)	*p*	OR (95%CI)	*p*	OR (95%CI)	*p*
Age	0.89 (0.66–1.12)	0.38	0.95 (0.75–1.18)	0.65	0.98 (0.85–1.14)	0.81	0.95 (0.86–1.00)	0.43
non-HPV16/18	0.69 (0.06–6.42)		1.31 (0.23–7.26)	0.74	1.21 (0.21–7.65)	0.83	1.05 (0.27–4.24)	0.94
Multiple HPV	0.08 (0.04–0.72)	0.045 *	0.12 (0.02–0.67)	0.026 *	0.42 (0.08–1.83)	0.27	0.30 (0.08–1.01)	0.06
Abnormal cytology	4.77 —	0.99	0.19 (0.02–1.26)	0.14	0.53 (0.06–2.86)	0.50	0.79 (0.17–3.02)	0.73
Transformation Zone	9.05 —	0.35	7.89 (0.42–9.36)	0.16	0.47 (0.40–4.8)	0.53	1.45 (0.24–8.55)	0.68
Lesion characteristics (CINIII)	—	0.99	0.09 (0.01–0.99)	0.07	0.34 (0.41–3.74)	0.36	0.26 (0.04–1.49)	0.14

* *p* < 0.05.

## Data Availability

The data presented in this study are available upon request from the corresponding author. The data are not publicly available due to privacy restrictions.

## Abbreviations

The following abbreviations are used in this manuscript:

HPV	human papillomavirus
CIN	cervical intraepithelial neoplasia
PDT	photodynamic therapy
CKC	cervical conization
CI	confidence interval
OR	odds ratio
HSIL	cervical high-grade squamous intraepithelial lesions

## References

[1] World Health Organization (2014). WHO Guidelines Approved by the Guidelines Review Committee. WHO Guidelines for Treatment of Cervical Intraepithelial Neo-Plasia 2–3 and Adenocarcinoma In Situ: Cryotherapy, Large Loop Excision of the Transformation Zone, and Cold Knife Conization.

[2] Ferrari F., Bonetti E., Oliveri G., Giannini A., Gozzini E., Conforti J., Ferrari F.A., Salinaro F., Tisi G., Ciravolo G. (2024). Cold Knife Versus Carbon Dioxide for the Treatment of Preinvasive Cervical Lesion. Medicina.

[3] Zhao X., Zhang R., Song S., Wang Y., Mu X. (2024). Analysis of the clinical characteristics and surgical methods of high-grade squamous intraepithelial lesions of the cervix in postmenopausal women: A retrospective case study. Medicine.

[4] Cui N., Li X., Wen X., Xu J., Chen L. (2024). Pathological Changes and Pregnancy Outcomes in Cervical Intraepithelial Neoplasia Patients After Cold Knife Conization. Int. J. Gen. Med..

[5] Wei Y., Gu L., Zhang Y., Yang Q., Chen F., Hong Z., Di W., Qiu L. (2025). Efficacy of ALA-PDT in treating cervical low-grade squamous intraepithelial lesions with high-risk HPV patients: A multicentre randomized controlled trial. Int. J. Cancer.

[6] de Castro C.A., da Silva C.F., Silva F.L., Marchetti L.O., Lombardi W., Bagnato V.S., Inada N.M. (2025). Evaluating photodynamic therapy protocols for high-grade cervical neoplasia: A comparative study. Photodiagn. Photodyn. Ther..

[7] Bodner K., Bodner-Adler B., Wierrani F., Kubin A., Szölts-Szölts J., Spängler B., Grünberger W. (2003). Cold-knife conization versus photodynamic therapy with topical 5-aminolevulinic acid (5-ALA) in cervical intraepithelial neoplasia (CIN) II with associated human papillomavirus infection: A comparison of preliminary results. Anticancer Res..

[8] Kapp P., Schmucker C., Siemens W., Brugger T., Gorenflo L., Röbl-Mathieu M., Grummich K., Thörel E., Askar M., Brotons M. (2025). Human papillomavirus (HPV) vaccination in women with conisation. Cochrane Database Syst. Rev..

[9] Fu K., Yangzom K., Li L., Wu L., Zhang Y. (2025). Optimizing the Follow-Up Interval After Successful Cold Knife Conization of CIN3: A 10-Year Retrospective Cohort Study. Cancer Med..

[10] McCluggage W.G., Singh N., Gilks C.B. (2022). Key changes to the World Health Organization (WHO) classification of female genital tumours introduced in the 5th edition (2020). Histopathology.

[11] Wang X., Qiu H., Zhan H., Huang Z. (2024). Photodynamic therapy of vaginal intraepithelial neoplasia-How to do it?. Photodiagn. Photodyn. Ther..

[12] Cai H., Che Y., Chen Y., Sun H., Ma T., Wang Y. (2024). Long-term follow-up of photodynamic therapy of cervical intraepithelial neoplasia grade 2 (CIN2). Photodiagn. Photodyn. Ther..

[13] López-Cárdenas M.T., Jiménez A., Espinosa-Montesinos A., Maldonado-Alvarado E., Osorio-Peralta M.O., Martinez-Escobar A., Moreno-Vázquez A., Aguilera-Arreola M.G., Ramón-Gallegos E. (2023). Elimination of Human Papillomavirus and Cervical Pathological Microbiota with Photodynamic Therapy in Women from Mexico City with Cervical Intraepithelial Neoplasia I. Photochem. Photobiol..

[14] Wang X., You L., Zhang W., Ma Y., Tang Y., Xu W. (2022). Evaluation of 5-aminolevulinic acid-mediated photodynamic therapy on cervical low-grade squamous intraepithelial lesions with high-risk HPV infection. Photodiagn. Photodyn. Ther..

[15] Qiu L., Li J., Chen F., Wang Y., Wang Y., Wang X., Lv Q., Li C., Li M., Yang Q. (2022). Chinese expert consensus on the clinical applications of aminolevulinic acid-based photodynamic therapy in female lower genital tract diseases (2022). Photodiagn. Photodyn. Ther..

[16] Wang X., Xu X., Ma Y., Tang Y., Huang Z. (2024). Comparative Study of 5-Aminolevulinic Acid-Mediated Photodynamic Therapy and the Loop Electrosurgical Excision Procedure for the Treatment of Cervical High-Grade Squamous Intraepithelial Lesions. Pharmaceutics.

[17] Tang Y., Su Y., Xu Y., Zhang Y., Shen Y., Qin L., Zhang L., Cao L., Zhou Y., Zhang T. (2022). Therapeutic effects of topical photodynamic therapy with 5-aminolevulinic acid on cervical high-grade squamous intraepithelial lesions. Photodiagn. Photodyn. Ther..

[18] Ma L., Gao X., Geng L., You K., Wu Z., Li Y., Han Q., Wang Y., Guo H. (2021). Efficacy and safety of photodynamic therapy mediated by 5-aminolevulinic acid for the treatment of cervical intraepithelial neoplasia 2: A single-center, prospective, cohort study. Photodiagn. Photodyn. Ther..

[19] Wang L., Liu X., Zhang J., Song M., Liu H., Xu Y., Meng L., Zhang Y., Jia L. (2024). Comparison of 5-ALA-PDT and LEEP of cervical squamous intraepithelial neoplasia (CIN2) with high-risk human papillomavirus infection in childbearing age women: A non-randomized controlled polit study. Photodiagn. Photodyn. Ther..

[20] Zhang T., Zhang Y., Tang Y., Qin L., Shen Y., Wang B., Zhang L., Cao L., Zhou Y., Su Y. (2022). The effect of high-risk HPV E6/E7 mRNA on the efficacy of topical photodynamic therapy with 5-aminolevulinic acid for cervical high-grade squamous intraepithelial lesions. Photodiagn. Photodyn. Ther..

[21] Ferrari F.A., Magni F., Bosco M., Biancotto G., Zorzato P.C., Laganà A.S., Chiantera V., Raffaelli R., Franchi M., Uccella S. (2023). The Role of Micronutrients in Human Papillomavirus Infection, Cervical Dysplasia, and Neoplasm. Healthcare.

[22] Cai L., Huang Y., Lin C., Liu G., Mao X., Dong B., Lu T., Sun P. (2020). A comparison study of post-operative infection analysis of cold-knife conization and loop electrosurgical excision procedure for cervical high-grade squamous intraepithelial lesion. Transl. Cancer Res..

[23] Bittencourt D.D., Zanine R.M., Sebastião A.P.M., Ribas C.M. (2023). Risk Factors for Persistence or Recurrence of High-Grade Cervical Squamous Intraepithelial Lesions. Rev. Col. Bras. Cir..

[24] Wang B., Yuan S., Su Y., Zhang C., Zhou M., Zhang M., Dai K., Wang Y., Cao L., Zhang T. (2024). Comparative study of topical 5-aminolevulinic acid photodynamic therapy and surgery for recurrent cervical high-grade squamous intraepithelial lesions following surgery. Photodiagn. Photodyn. Ther..

